# First molecular-cytogenetic characterization of Fanconi anemia fragile sites in primary lymphocytes of FA-D2 patients in different stages of the disease

**DOI:** 10.1186/s13039-016-0280-6

**Published:** 2016-09-13

**Authors:** Jelena Filipović, Gordana Joksić, Dragana Vujić, Ivana Joksić, Kristin Mrasek, Anja Weise, Thomas Liehr

**Affiliations:** 1 Vinca Institute of Nuclear Sciences, University of Belgrade, Mike Petrovica Alasa 12-14, Belgrade, 11001 Serbia; 2 Mother and Child Health Care Institute of Serbia, “Dr Vukan Cupic”, Radoja Dakica 6, Belgrade, 11070 Serbia; 3 Institute of Human Genetics, Jena University Hospital, Friedrich Schiller University, Kollegiengasse 10, Jena, D-07743 Germany

**Keywords:** Fanconi anemia, Fragile sites, Telomere fusions, Radial figures, X chromosome

## Abstract

**Background:**

Fanconi anemia (FA) is a chromosomal instability syndrome characterized by increased frequency of chromosomal breakages, chromosomal radial figures and accelerated telomere shortening. In this work we performed detailed molecular-cytogenetic characterization of breakpoints in primary lymphocytes of FA-D2 patients in different stages of the disease using fluorescent in situ hybridization.

**Results:**

We found that chromosomal breakpoints co-localize on the molecular level with common fragile sites, whereas their distribution pattern depends on the severity of the disease. Telomere quantitative fluorescent in situ hybridization revealed that telomere fusions and radial figures, especially radials which involve telomere sequences are the consequence of critically shortened telomeres that increase with the disease progression and could be considered as a predictive parameter during the course of the disease. Sex chromosomes in FA cells are also involved in radial formation indicating that specific X chromosome regions share homology with autosomes and also could serve as repair templates in resolving DNA damage.

**Conclusions:**

FA-D2 chromosomal breakpoints co-localize with common fragile sites, but their distribution pattern depends on the disease stage. Telomere fusions and radials figures which involve telomere sequences are the consequence of shortened telomeres, increase with disease progression and could be of predictive value.

## Background

Fanconi anemia (FA) is a rare, inherited, genetically heterogeneous chromosomal instability syndrome. To date, 19 different complementation groups, which correspond to distinct DNA repair genes have been identified, FA-A, FA-B, FA-C, FA-D1 (*BRCA2*), FA-D2, FA-E, FA-F, FA-G, FA-I, FA-J (*BRIP1*), FA-M, FA-N (*PALB2*), FA-O (*RAD51C*), FA-P (*SLX4*), FA-Q (*ERCC4*), FA-S (*BRCA1*), FA-R (*RAD51*) and FA-T (*UBE2T*) [1]. The genes that correspond to different FA complementation groups are involved in the FA/BRCA DNA damage repair pathway having an essential function in the cellular response to stress induced by DNA alkylating agents [2]. The most frequent complementation group in Serbia is FA-D2 [3], which is in contrast to the FA-D2 frequency in the general population where it accounts for approximately 4 % of all complementation groups [4]. FA-D2 patients are characterized by a broad spectrum of congenital abnormalities and, unlike most of the other FA complementation groups, an early onset of hematological manifestations including acute myelogenous leukemia and bone marrow failure [5]. One of the most significant FA cellular characteristics is an increase of chromosomal aberrations induced by interstrand cross-linking (ICL) agents [6]. When exposed to these agents, FA cells show an increased frequency of chromosomal breakages and chromosomal radial figures, prolonged cell-cycle arrest in G2/M phase, reduced cellular survival and accelerated telomere shortening [7]. With respect to chromosomal breakages, Schoder and coworkers [8] reported that chromosomal breakpoints in cells derived from FA-A and FA-C patients co-localize with the chromosomal regions that are constitutionally prone to breakage in each individual, known as common fragile sites (CFSs). In addition to standard FS classification, it was shown that cells exposed to replication stress also display breakages in telomeric regions; therefore they were attributed to CFSs [9]. Recent studies of telomere maintenance in FA cells showed that telomere fusions arise either as a consequence of their critical shortening or altered capping function. High percentage of dysfunctional telomeres and marked telomere fragility was found to be typical for FA-D2 cellular phenotype [10]. However, the role of telomeres in formation of induced chromosomal aberrations such as radial figures has not been determined, yet.

The aim of this study was to provide the first molecular-cytogenetic characterization of breakpoint distribution and co-localization with FSs, formation of radial figures and involvement of telomeres in formation of chromosomal aberrations in FA-D2 primary lymphocytes originated from patients in different stages of the disease, severe and mild bone marrow failure stage.

## Methods

### Patients and sampling

A total of six patients (five females, one male) previously diagnosed with FA-D2 [3, 10] were included in our study. Mean age of children at the time of study was 8 ± 5 years. Routine control of those children was undertaken at the Mother and Child Health Care Institute of Serbia, Hematooncology department. Disease stage was determined based on complete blood count (CBC) and bone marrow examination using standard criteria. Children were divided into two groups accordingly: severe (group A) or mild (group B) bone marrow failure (BMF). Informed consents from the families were obtained and peripheral blood was collected in Li-heparin vaccutainers for additional testing. The study was approved by The Ethical Committee of Mother and Child Health Care Institute of Serbia.

### Whole blood cultures

Aliquots of heparinized whole blood (0.5 ml) were set up in cultures containing PBmax-karyotyping medium (Invitrogen-Gibco, Paisley, UK) and treated with diepoxibuthan (DEB, Sigma Chemicals Co., Germany) (final concentration 0.1 μg/ml) 48 h after culture initiation and further harvested 72 h after initiation. Colchicine (Sigma-Aldrich, Munich, Germany) was added during the last 3 h (final concentration 2.5 μg/ml). Cells were collected by centrifugation and treated with hypotonic solution (0.56 % KCl). Cell suspensions were fixed in methanol/acetic acid (3:1), washed three times with fixative and dropped onto clean slides.

### DAPI banding

Slides were incubated overnight at room temperature, dehydrated in series of ethanol (70, 95 and 100 %, respectively), 5 min each, and counterstained with 4′,6′-diamidino-2-phenylindole (DAPI)-containing Vectashield solution (Vector Laboratories Ltd, Peterborough, UK). DAPI selectively binds to heterochromatin regions, which produces a banding pattern known as inverse DAPI banding. According to the unique pattern of differentially stained regions each chromosome can be identified. Analysis was performed with a Zeiss-Axioimager A1 microscope and the ISIS imaging software package (MetaSystems, Altlussheim, Germany).

### Locus-specific fluorescent in situ hybridization (Locus-specific FISH, BAC-FISH)

After determination of the most frequent chromosomal breakpoints by inverse DAPI staining, molecular characterization was performed by hybridization with appropriate bacterial artificial chromosome probes (BAC-probes) as described by Mrasek et al. [11]. In short, after removing DAPI, slides were incubated in pepsin solution for 5 min, washed in 1xPBS and fixed in 1 % formalin buffer solution. Prior to applying the BAC-probe, slides were dehydrated in series of ethanol (70, 90, 100 %, 5 min each), denatured in 70 % formamide solution for 2–3 min on a hot plate at 75 °C and immediately placed in 70 % ethanol (4 °C) to conserve target DNA single-stranded. Dehydration in 90 and 100 % ethanol followed. The probe mixture (the probe in hybridization buffer and COT1 Human DNA) was denatured in a thermocycler at 75 °C for 5 min, pre-annealed at 37 °C for 30 min and cooled down to 4 °C. Prepared denatured probes were applied to the slides and hybridized overnight in a dark humid chamber at 37 °C. After hybridization, the slides were washed in 4xSSC/0.2 % Tween20 at the room temperature and 1xSSC-solution (62–65 °C), dehydrated in series of ethanol, counterstained with DAPI solution and observed under the fluorescence microscope. The results were analyzed using ISIS software (MetaSystems, Germany). The chromosomal regions and associated BAC-probes used for hybridization are shown in Table 1.

**Table 1:** BAC-probes used for fluorescence in situ hybridization for the most breakpoint-affected chromosomal regions

Chromosomal region	Fragile site (FS)	FS type^a^	BAC-probe	Co-localization of FA breakpoints and FS
1p13.3	FRA1N	Aphidicolin^b^	RP11-242D10	+
1q21.2	FRA1F	Aphidicolin	RP11-301 M17	+
1q42.2	Near FRA1H	Azacytidin	RP11-109G24	−
2q35	FRA2U	Aphidicolin	RP11-316O14	+
3p14.2	FRA3B	Aphidicolin	RP11-129 K20	+
3p21.31	FRA3H	New nomenclature^c^	RP11-787O14 RP11-159A17	+
3q27.1	FRA3C	Aphidicolin	RP11-110C15	+
5q13.2	FRA5K	New nomenclature^d^	RP11-497H16	+
5q33.2	FRA5O	New nomenclature^d^	RP11-265I24 RP11-494C5	+
7q22.3	FRA7F	Aphidicolin	CTB-20D2	+
7q32.3	FRA7H	Aphidicolin	RP11-138A9 RP11-36B6	+
11q13	FRA11H	Aphidicolin	RP11-449G14	+
14q24.3	FRA14G	Aphidicolin^b^	RP3-414A15	+
16q22.1	FRA16C, FRA16B	Aphidicolin	RP11-106 J23	+
16q23	FRA16D	Aphidicolin	RP11-358 L22	+
18q21.3	FRA18B	Aphidicolin	RP11-15C15	+

BAC-probes for the appropriate FSs were selected according to the literature data:

^a^Nomenclature according to Lukusa et al. [33]

^b^New extended nomenclature according to Mrasek et al. [34]

^c^Common according to the nomenclature by Borgaonkar [35]

^d^Common according to the nomenclature by Simonic and Gericke [36]

### Telomere quantitative fluorescent in situ hybridization (Q-FISH)

After metaphase analysis DAPI was removed, slides hybridized with telomere cPNA oligonucleotide probe (TTAGGG) as described by Slijepcevic [12]. Briefly, hybridization was performed with the Cy-3 labeled telomeric PNA probe (CCCTAA) 3′ supplemented with PNA centromeric probe for chromosome 2 (DAKO, Glostrup, Denmark) in final concentration 2 ng/ml. The PNA probe for centromere 2 was added in ready to use telomere probe. After hybridization slides were left in a dark humidified chamber for 2 h. The slides were then washed in 70 % formamide and stained with 4′,6′-diamidino-2-phenylindole (DAPI)-containing mounting medium (Vector Laboratories, UK). Analysis was performed using the ISIS software, MetaSystems (Altlussheim, Germany). Measurements were reported as arbitrary relative telomere length units (RTLU), which are defined as the ratio of signal intensity between telomeres and a centromere chromosome 2 reference signals.

### Metaphase analysis

Metaphase spreads were analyzed according to the International System for Human Cytogenetic Nomenclature (ISCN, 2013). For each sample at least 1000 metaphases were analyzed for the breakpoint characterization, whereas at least 500 metaphases were scored for fusions and radial formation with regard to the chromosomes involved in their formation, and involvement of telomeres in arising of those aberrations.

### Statistical analysis

FA breakpoints were expressed as the percentage of total number of breaks per patient. Telomere fusions and radial figures were presented as percentage of total number of metaphases analyzed and were statistically analyzed using nonparametric Mann-Whitney *U* test in the program SPSS 10 for Windows. Differences at *p* < 0.05 were considered as significant.

## Results

### FA breakpoints and assignment to FSs

Results of molecular cytogenetic characterization concerning chromosomal breakages, telomere fusions and radial figures in DEB treated peripheral blood derived from three FA-D2 patients in the severe stage of diseases (group A), and three patients in the mild stage of the disease (group B) are presented in Table 2.

**Table 2:** FA-D2 patients’ karyotypes and molecular cytogenetic findings

	FA-D2 patient	Age (years)	Karyotype	Most frequent breakpoints	Fragile site (FS)	Breakpoint frequency (%)	Telomere fusion frequency (%)	Average telomere length (RTLU^a^ ± SD)	Radial figures frequency (%)
Group A	1	8	46,XX	1q42.2	distal to FRA1H	1.72	1.95	14.76 ± 3.23	7.06
				14q24.3	FRA14G	3.09			
				18q21.3	FRA18B	1.72			
	2	6	46,XX	2q35	FRA2U	2.4	1.54	23.02 ± 12.73	1.78
				3p14.2	FRA3B	2.4			
				5q33.2	FRA5O	2			
				7q32.3	FRA7H	1.6			
	3	17	46,XX	3q27.1	FRA3C	2.75	1.62	35.72 ± 7.00	1.88
				5q13.2	FRA5K	2.75			
				5q33.2	FRA5O	3.29			
Group B	4	3	46,XX	3p14.2	FRA3B	11.07	0.08	20.48 ± 12.24	0.78
				3p21.31	FRA3H	3.95			
				7q32.3	FRA7H	1.19			
	5	3	46,XX,der(21)t(15;21)(q15;p11.1)[5]/46,XX [15]	1p13	FRA1N	1.76	0.72	21.01 ± 10.33	0.715
				1q21	FRA1F	1.32			
				3p21.31	FRA3H	1.32			
				11q13	FRA11H	0.88			
	6	11	46, XY, der(16)t(1;16)(q42;p11.2)del(1) (q42)[1]/46,XY[31]	3p14.2	FRA3B	1.88	0.23	23.91 ± 6.92	0.23
				3p21.31	FRA3H	0.63			
				16q22.1	FRA16C	1.88			
				16q23	FRA16D	6.88			

^a^RTLU–relative telomere length units

**Fig. 1:**
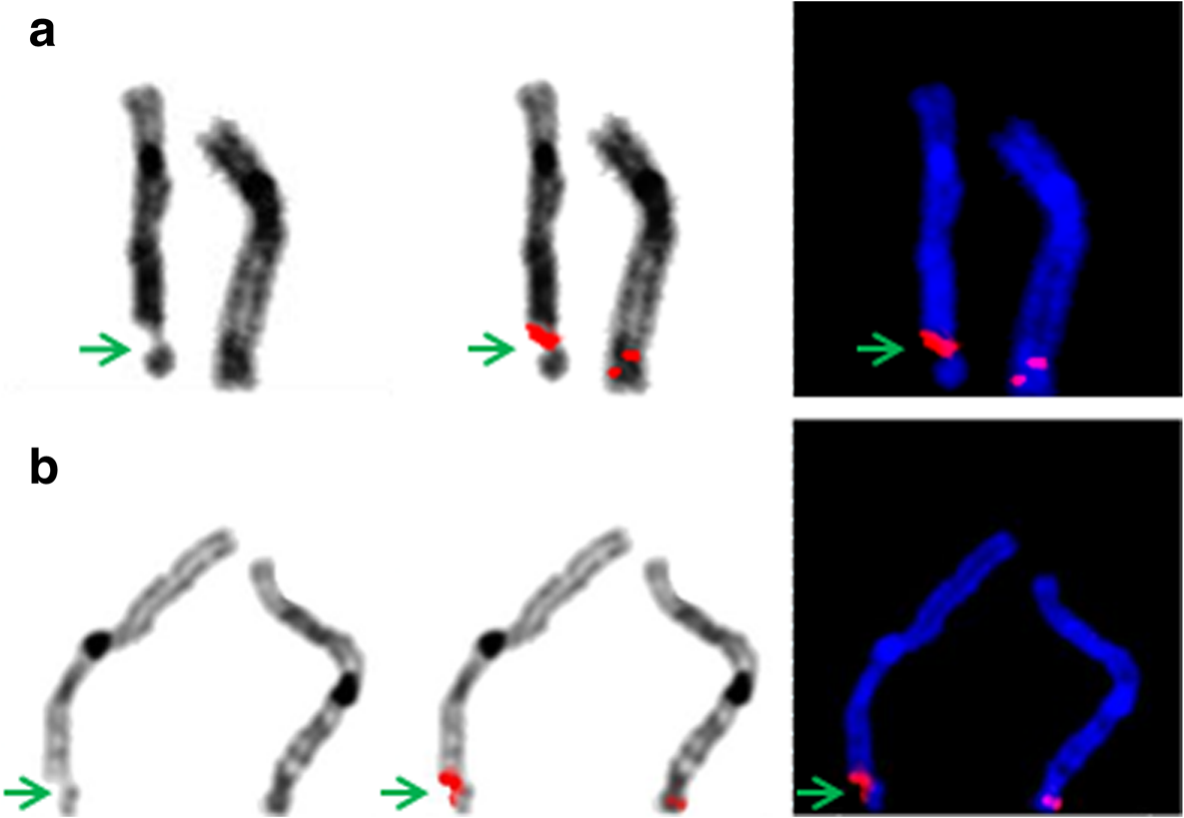
Representative images of BAC-FISH in FA-D2 metaphases, inverted DAPI and DAPI staining. Arrows indicate breakpoints and hybridized BAC-probes; RP11-265I24 located within FRA5O (**a**), and RP11-109G24 located within 1q42.2, near FRA1H (**b**)

The FA breakpoint analysis showed that frequency of breakages, and breakpoint distribution pattern depend on the stage of the disease: most of the breakpoints in both groups of patients corresponded to CFSs, except for patient 1 in group A where one of the most frequent breaks was 1q42.2 (Fig. 1). In the group A patients, breakpoints were dispersed throughout the genome with a much lower frequency of breakage at the particular chromosomal locus, i.e. the most frequent breakpoints within this group did not exceed 3.297 % of the total number of breaks.

In contrast, chromosomes in patients of B group tended to break with the higher frequency in the chromosomal regions where the most CFSs are reported (Table 2). Interindividual variability in frequency of the most common breakages was significant in this group, but the breakage pattern in two patients from this group was similar: in patients 4 and 6 the highest frequency of breakage was observed in regions that correspond to FRA3B (11.07 %) and FRA16D (6.87 %), respectively. However, patient 5 had a different breakage pattern compared to other two patients within this group, where low frequencies of the common breakages were found. However, this patient also shows an unbalanced karyotype, mos 46,XX,der(21)t(15;21)(q15;p11.1)[5]/46,XX[15].

### Telomeres and radial figures

**Fig. 2:**
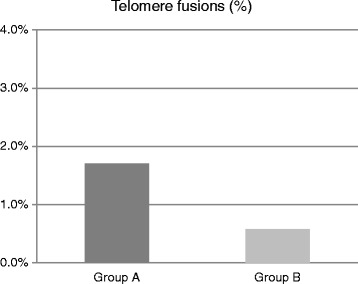
Percentage of telomere fusions in group A and B patients. Group A display more fusions than group B

**Fig. 3:**
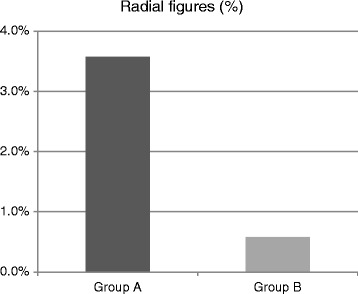
Percentage of radial figures in group A and B patients. In Group A incidence of radials was six fold higher compared to group B

Frequency of telomere fusions and radial figures, with special regard to their structure and telomere involvement in radial figures formation, are presented in Table 2 and Figs. 2, 3 and 6.

**Fig. 4:**
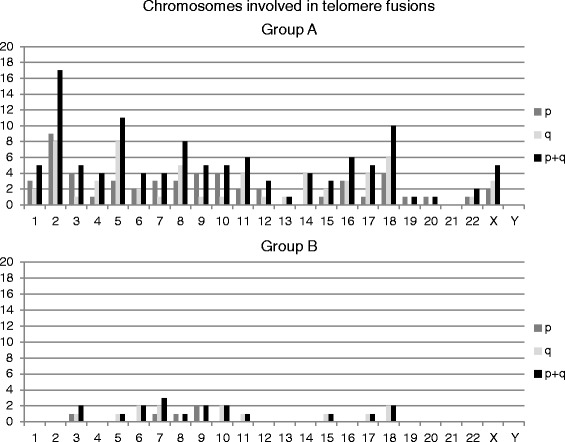
Chromosomes involved in telomere fusions in group A and B

The results showed significantly higher frequency of telomere fusions in patients from group A versus group B (*p* = 0.05, Fig. 2). Within A group of patients, telomere fusions occurred most frequently between chromosomes 2p, 2q, 5q and 18q, whereas in group B fusions mostly occurred between 6q, 7q, 9p, 10q, 18q (Fig. 4). Relative quantification of telomere length revealed that the average telomere length (RTLU) was not significantly different between two groups (*p* = 0.442). However, quantification of telomere length at single chromosomes showed that p/q ratio ranged from 0.41 to 2.18 and that chromosomes with the shortest telomeres constituted telomere end-to-end fusions.

**Fig. 5:**
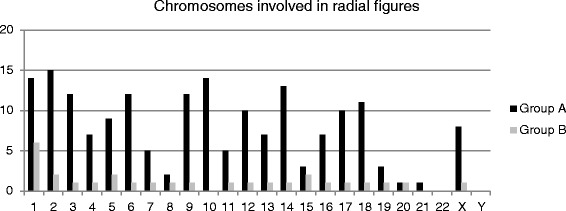
Chromosomes involved in radial figures in both patient groups

**Fig. 6:**
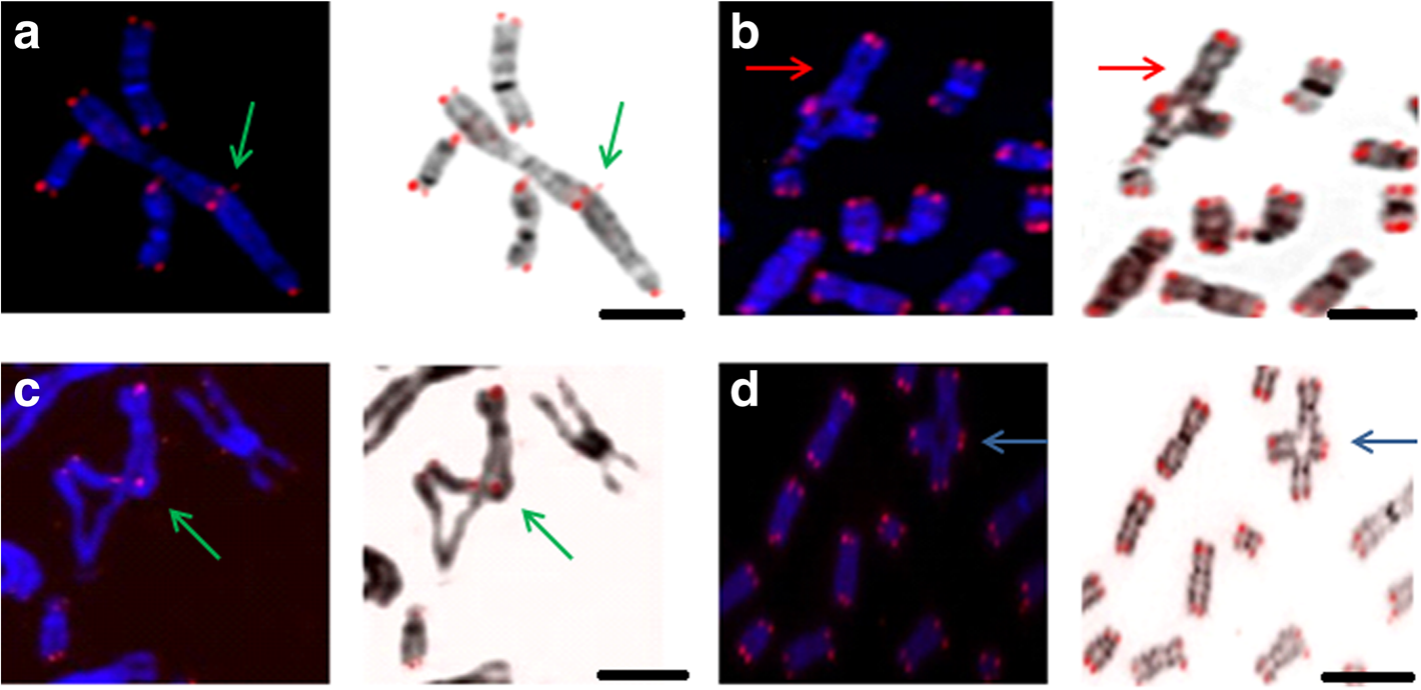
Representative images of telomere FISH in FA-D2 metaphases. Telomere signals at the fusion points are indicated by *green arrow*: telomere end-to-end fusion, three signals are present between two chromosomes (**a**), radial figure between chromosomes X and 13, *red arrow* indicates X chromosome in radial figure (**b**), *green arrow* indicates radial figure between telomere of one and interstitial region of another chromosome (**c**), *blue arrow* indicates simple radial figure (**d**). Scale bars = 10 μm

The percentage of radial figures was low in group B (below 1 %), whereas in group A the percentage was on average six times higher than in group B (*p* = 0.05, Fig. 3). Regarding radial figure structure, great percentage of radials found in group A was a consequence of exchange between telomeres of one and non-telomeric, interstitial regions of other chromosomes, 25.49 % (50 % of all radials in patient 1, and 22.7 % for patients 2 and 3, each). Only two radials between telomeres and interstitial chromosomal sequences were observed in patient 4 and 5, and neither one was observed in patient 6. Although radials always arose between non-homologous chromosomes, distribution of involved chromosomes was heterogeneous–chromosomes 1, 2, 9, 10 and 14 were frequently present in radial figures in group A patients, and chromosome 1 was most frequent in group B. Chromosome X was found to be involved in radial figures in all group A patients and one group B patient (Fig. 5). Representative images of telomere fusions, radials and X chromosome in radial formation are presented in the Fig. 6.

## Discussion

The results of our study revealed that chromosomes are not equally prone to breakages in FA-D2 cells exposed to DEB. Matching the data of DAPI banding and BAC-FISH pointed out that regions with increased frequency of breakages co-localize with the CFSs, indicating that frequency of breakpoints as well as their distribution pattern is different among patients and is related to the disease stage.

Assignment of breakpoints to FS in FA-D2 cells is in accordance to previously reported data for FA-A and FA-C cell lines, although the disease stage for the patients in this study was not determined [8]. In FA-D2 patients in the severe stage of the disease (group A) the most frequent breakpoints were present within the CFSs regions (FRA14G, FRA5O, FRA5K and FRA3C, Table 2) except for patient 1 in group A where one of the most frequent breaks was 1q42.2 which is near the FRA1H [13]. However, previously it was not attributed as FS. Their distribution pattern was similar to that found in FA-A and FA-C patients, although different breakpoints were observed in these groups (FRA1A, FRA1D, FRA1E and FRA1J) [8]. The average incidence of chromosomal breaks didn’t increase with the disease progression, but the breakpoint distribution pattern was quite different. Mild stage patients (group B) displayed higher frequencies of breakpoints within the most common FSs, FRA3B and FRA16D except for the patient with unbalanced karyotype (Table 2).

FA pathway plays an essential role in regulation of maintenance and stability of CFSs [14]. Howlett et al. [15], was the first who establishes that disrupted FA pathway leads to increased chromosomal instability at CFSs underlying that their stability depends on the ATR-FANCD2 signaling pathway, i.e. following DNA replication stress, ATR kinase phosphorylates FANCD2 promoting its monoubiqutination, which activates homologous recombination (HR). Replication stress leads to accumulation of FANCD2 protein in the chromosomal regions prone to breakage, whether they are broken or not [16]; however altered FANCD2 protein in FA-D2 cells couldn’t maintain stability of existing FSs which in turn activates alternative mechanisms of repair. Apart from HR the CFSs are maintained by non-homologous end-joining (NHEJ) mechanisms, as well as with specialized polymerases that perform by-pass function in DNA synthesis [17, 18].

On the other hand, CFSs are AT-rich sequences prone to form DNA secondary structures which can lead to the replication fork collapse [19]. In addition, replication timing is delayed along CFSs, whereas exposure to DNA polymerase inhibitors intensifies the deceleration of the fork progression, so the CFSs enter G2 phase unreplicated [20]. Both give rise for chromosomal breaks.

Increasing body of evidence now show that large genes constitute the pool of CFSs [21] indicating relationship between instability and transcription of these genes. Since more than one cell cycle is required for their transcription, transcription and replication machineries collide and CFSs expression may occur [22]. Although different FS distribution pattern with the disease progression remains unclear, it is possible that with the progression of the disease different genes could be expressed as a cellular attempt to compensate progressive genomic instability, leading to different CFSs expression.

Up to now, 19 CFSs have been molecularly characterized [23]. Among them are the most common FSs (FRA3B and FRA16D, Table 3) which both contain very large genes (>1 Mbp) important for tumor suppression. These FSs are, along with FRA7H, most frequently expressed in mild stage. In severe stage of the disease different FSs appear as the most common. These chromosomal regions are also very large, extending from 2 to 6 Mbp, but are comprised by a great number of genes (Table 3); many of those are included in control of cell cycle and tumor suppression. For example, *BCL2* gene (spans approximately 200 kbp), which is the first discovered anti-apoptotic gene, is located within FRA18B, whereas it is known that breakpoints and translocations within this region disrupt its function and lead to myeloproliferative diseases [24]. Similarly, protooncogene *C-FOS*, located within FRA14G, also has important role in regulation of cell proliferation and differentiation [25].

**Table 3:** Genes located within common fragile sites according to the literature data^a, b, c, d^

Fragile site	Chromosomal region	Start	End	Gene	Gene length
Molecularly characterized fragile sites					
FRA3B	3p14.2	58 600 001	63 800 000	FHIT^a^	>1.5 Mbp
FRA7H	7q32.3	130 800 001	132 900 000	/^b^	/
FRA16D	16q23.2	79 200 001	81 600 000	WWOX^c^	1.1 Mbp
Not-characterized fragile sites^d^					
FRA1F	1q21.2	147 500 001	150 600 000	33 genes	/
FRA1N	1p13.3	106 700 001	111 200 000	52 genes	/
FRA2U	2q35	214 500 001	220 700 000	70 genes	/
FRA3C	3q27.1	183 000 001	184 800 000	20 genes	/
FRA3H	3p21.31	44 200 001	50 600 000	78 genes	/
FRA5K	5q13.2	69 100 001	74 000 000	25 genes	/
FRA5O	5q33.2	153 300 001	156 300 000	8 genes	/
FRA7F	7q22.3	104 900 001	107 800 000	14 genes	/
FRA11H	11q13.4	70 500 001	75 500 000	48 genes	/
FRA14G	14q24.3	73 300 001	78 800 000	69 genes	/
FRA16C	16q22.1	66 600 001	70 800 000	95 genes	/
FRA18B	18q21.3	61 300 001	63 900 000	10 genes	/

^a^According to Zimonjic et al. [37]

^b^According to Mishmar et al. [38]

^c^According to Ried et al. [39]

^d^According to National Center for Biotechnology Information [40]

Patients in the severe stage of the disease have significantly higher percentage of telomere fusions compared to the patients in the mild stage. Interestingly, there was no significant difference in average telomere length between two groups of patients, but measurement of telomere length at each individual chromosome revealed that the chromosomes with the shortest telomeres were most frequently involved in telomere fusions. Our previous research showed that lymphocyte telomeres in FA-D2 patients are shortened when compared to the age-matched control [10]. Taking into account that FA cells are breakage prone, increased breakages at telomeres and complexity of their function could be the cause of their shortening. Recent review of Holohan et al. [26] pointed out that impaired telomere maintenance and attrition is a hallmark of FA-D2 group, not FA in general. Our previous study showed that FA-D2 cells displayed heterogeneous telomere length and high frequency of double-strand breaks in telomere regions (telomere dysfunction–induced foci - TIFs) which lead to telomere fragility [10]. Altered FANCD2 protein is not capable to maintain telomeres, leaving the telomeres unprotected and prone to increased fragility and attrition.

FA-D2 patients in the severe stage had significantly more radials than the patients in the mild phase of the disease, particularly radials formed between telomeres of one and interstitial chromosomal regions of other chromosomes, which was rarely observed in group B. This is unreported finding. Since shortened and fragile telomeres act as double strand breaks (DSB) interchromosomal recombination with other impaired chromosomal regions in attempt to repair the damage is not surprising. Additionally, two patients in severe stage developed bone marrow failure several months after cytogenetic examination and became candidates for bone marrow transplantation.

In both groups of patients, radials were composed of non-homologous chromosomes, which is consistent with the previously reported results [27, 28]. Distribution of involved chromosomes was heterogeneous and not specific for the disease stage. However, X chromosome was found in radials in both groups of patients, which is opposite to the report of Newell and colleagues [27], who found that sex chromosomes are not involved in FA-A and FA-G cells and suggested that alternative mechanisms of ICL repair, which avoid recombination between sex and autosome chromosomes, play the main repair role in FA cells. Involvement of Y chromosome was not observed, but only one male patient in the mild stage and with very low number of radials was part of the study. The presence of sex chromosomes in radial figures in FA patients was not previously reported.

Although radials are described in many genomic instability syndromes, only a few studies clarified the mechanism of their formation. Kuhn and Therman [29] and Scully et al. [30], suggested that they arise as an attempt of cellular repair machinery to resolve DSBs, especially when cells with hampered repair machinery are exposed to ICL-s employing homologous recombination [31, 32]. Radials formation in FA cells between non-homologous chromosomes, as well as between autosomes and sex chromosomes indicate that short dispersed chromosomal regions that share homology could act as repair templates in resolving the DNA damage. In FA-D2 cells the X chromosome is not spared from such recombination.

## Conclusions

Overall, our results indicate that regions with increased frequency of breakage co-localize with CFSs, whereas their distribution pattern relates to the severity of the disease.

Telomere fusions, as well as interchromosomal recombination and radials formation which involve telomere sequences are the consequence of critically shortened telomeres, increase with disease progression and could be of predictive value.
